# Comparative Analysis of Novel Strains of Porcine Astrovirus Type 3 in the USA

**DOI:** 10.3390/v13091859

**Published:** 2021-09-17

**Authors:** Franco Matias Ferreyra, Karen Harmon, Laura Bradner, Eric Burrough, Rachel Derscheid, Drew R. Magstadt, Alyona Michael, Marcelo Nunes de Almeida, Loni Schumacher, Chris Siepker, Panchan Sitthicharoenchai, Gregory Stevenson, Bailey Arruda

**Affiliations:** Veterinary Diagnostic Laboratory, Department of Veterinary Diagnostic and Production Animal Medicine, Iowa State University, 1850 Christensen Drive, Ames, IA 50011-1134, USA; funquillo@hotmail.com (F.M.F.); kharmon@iastate.edu (K.H.); lbradner@iastate.edu (L.B.); burrough@iastate.edu (E.B.); rdersch@iastate.edu (R.D.); magstadt@iastate.edu (D.R.M.); avdonina@iastate.edu (A.M.); malmeida@iastate.edu (M.N.d.A.); llschum@iastate.edu (L.S.); csiepker@iastate.edu (C.S.); psitthi@iastate.edu (P.S.); stevengw@iastate.edu (G.S.)

**Keywords:** mamastrovirus 22, porcine astrovirus type 3, polioencephalomyelitis, untranslated region, VPg: UTR, pseudoknot

## Abstract

Porcine astrovirus type 3 (PoAstV3) has been previously identified as a cause of polioencephalomyelitis in swine and continues to cause disease in the US swine industry. Herein, we describe the characterization of both untranslated regions, frameshifting signal, putative genome-linked virus protein (VPg) and conserved antigenic epitopes of several novel PoAstV3 genomes. Twenty complete coding sequences (CDS) were obtained from 32 diagnostic cases originating from 11 individual farms/systems sharing a nucleotide (amino acid) percent identity of 89.74–100% (94.79–100%), 91.9–100% (96.3–100%) and 90.71–100% (93.51–100%) for ORF1a, ORF1ab and ORF2, respectively. Our results indicate that the 5′UTR of PoAstV3 is highly conserved highlighting the importance of this region in translation initiation while their 3′UTR is moderately conserved among strains, presenting alternative configurations including multiple putative protein binding sites and pseudoknots. Moreover, two predicted conserved antigenic epitopes were identified matching the 3′ termini of VP27 of PoAstV3 USA strains. These epitopes may aid in the design and development of vaccine components and diagnostic assays useful to control outbreaks of PoAstV3-associated CNS disease. In conclusion, this is the first analysis predicting the structure of important regulatory motifs of neurotropic mamastroviruses, which differ from those previously described in human astroviruses.

## 1. Introduction

Astroviruses are a diverse group of positive-sense, single-stranded RNA viruses belonging to the family *Astroviridae,* order *Stellavirales*; and are classified into the genera *Mamastrovirus* and *Avastrovirus,* depending upon if they infect mammals or avian species, respectively [1]. Historically, members of both genera were frequently associated with cases of enteric disease and seldom associated with extraintestinal manifestations [2,3,4,5]. In the last decade, members of the genus *Mamastrovirus* have been increasingly associated with central nervous system (CNS) disease [6]. In 2010, both human and mink neurotropic strains were associated with cases of encephalomyelitis [3,7]. More recently, multiple neurotropic strains of astroviruses have been associated with cases of CNS disease affecting wild and domesticated animals including swine, sheep, cattle, muskox, and alpacas [8,9,10,11,12,13].

In humans, as in animals, cases of astrovirus-associated CNS disease are often fatal. Of the numerous neurotropic strains identified infecting mammals, only a couple strains infecting humans have been successfully isolated in cell culture [14,15]. This suggests that neurotropic strains belonging to different genogroups could use different strategies for virus replication, including various mechanisms for translation initiation [16]. Multiple research groups have unsuccessfully pursued the isolation of neurotropic mamastroviruses from clinical samples, and while this approach is still largely advantageous, in silico molecular studies may aid to elucidate unknown viral sequence motifs and mechanisms associated with a diverse range of biological activities for viruses difficult to isolate in cell culture systems [17,18,19].

The organization of the open reading frames (ORF), namely ORF1a, ORF1b, and ORF2, encoding the non-structural protein 1a (nsp1a), non-structural protein 1ab (nsp1ab), and the capsid protein, respectively, of the family *Astroviridae* is one of the most distinctive features of its members [20]. This arrangement, where structural proteins are encoded immediately after the 5’ untranslated region (UTR) distinguishes the particular genomic organization of astroviruses from other single-stranded RNA viruses affecting mammals.

Porcine astroviruses are a polyphyletic group of viruses and genetic differences of the complete capsid sequence (ORF2, adjacent to the 3′UTR) defines the current taxonomy. The taxonomy of porcine astroviruses has not been completely updated since the 9th report of the International Committee on Taxonomy of Viruses (ICTV) nearly a decade ago. Porcine astroviruses have been previously classified into seven genotype species (i.e., MAstV 3, MAstV 22, MAstV 24, MAstV 26, MAstV 27, MAstV 31, and MAstV 32), divided into five distinct genetic lineages (i.e., PoAstV1-5). All porcine astroviruses were initially identified in fecal samples, associated or not with cases of diarrhea; however porcine astroviruses have been recently associated with extraintestinal manifestations [21,22,23,24,25].

In 2018, strains of porcine astrovirus type 3 (PoAstV-3, tentatively *Mamastrovirus* 22) were associated with CNS disease affecting swine herds in both the USA and Hungary [12,26,27]. Subsequent studies have shown that PoAstV3 has been associated with cases of polioencephalomyelitis as early as 2010, with continued diagnosis in the US swine herd [28]. Retrospective data from multiple veterinary diagnostic laboratories in the USA indicate an increased detection of cases of PoAstV3 with 38 new cases of disease identified between December of 2017 and March 2021 [29,30]. In addition, experimental reproduction of disease with PoAstV3 has been recently demonstrated by inoculation of CNS tissue homogenate in colostrum-deprived, cesarean-derived (CDCD) piglets [31]. Although there have been multiple advances in the epidemiology and pathophysiology of PoAstV3, the virus has not been yet isolated in cell culture systems, hampering its research [19,22,32].

Previously, in silico analyses have modeled and identified important motifs for replication of astroviruses, including the frameshift signal, both UTRs, and multiple stem loops within their 3′ termini of their genome [33,34,35]. Additionally, multiple putative RNA-protein interaction regions have been identified with this approach in both UTRs of human astroviruses suggesting their potential association in multiple viral and host cell processes. Potential RNA-binding proteins (RBPs) previously identified included multiple serine/arginine-rich splicing factors (SRSF), heterogeneous nuclear ribonucleoprotein E2 (hnRNPE2), and polypyrimidine tract-binding protein (PTB or hnRNPI) [36]. Furthermore, recent studies comparing multiple human and animal astroviruses have identified a functional ORF, encoding a viroporin, and previously suggested by in silico analyses [17,37,38]. These studies and other studies demonstrate the usefulness of in silico analysis [39,40].

Due to the continued relevance of PoAstV3-associated polioencephalomyelitis in the US swine herd, we describe the comparative analysis of multiple novel strains of PoAstV3; focusing on the secondary RNA structure and motifs present in their untranslated regions and its association with RBPs; the presence of a putative VPg within its genome, and the identification of multiple linear epitopes and antigens within the capsid protein. This study predicts and describes multiple conserved motifs and potential molecules relevant to the life cycle and replication of PoAstV3, which may be important for its propagation in vitro and future studies involving neurotropic mamastroviruses. Furthermore, highly conserved epitopes and antigens identified in this study may allow us to develop diagnostic assays and vaccine candidates useful for the prevention of outbreaks associated with neurotropic strains of PoAstV3.

## 2. Materials and Methods

### 2.1. Diagnostic Samples

A subset of swine cases diagnosed with PoAstV3-associated polioencephalomyelitis at the Iowa State University Veterinary Diagnostic Laboratory (ISU-VDL) from January 2017 to December 2020 was included in this study (*n* = 64). Inclusion criteria included cases with histologic lesions consistent with a CNS viral infection (lymphoplasmacytic polioencephalomyelitis, perivascular cuffing, gliosis and neuronal necrosis), and concurrent detection of PoAstV3 by reverse transcription quantitative polymerase chain reaction (RT-qPCR) in CNS tissues. Additionally, feces (*n* = 2) and one intestinal tissue homogenate sample from farms with intermittent PoAstV3-associated polioencephalomyelitis were included.

### 2.2. RT-qPCR and Sanger Sequencing

For each diagnostic case, tissue homogenates used at the time of initial diagnosis were retrieved and stored at −80 °C. Previously, CNS tissues were processed as follow: aliquots (1 to 3 g) of CNS tissue were minced with sterile forceps and scissors, homogenized with 15 ml Minimal Essential media (MEM, Fisher Scientific, Waltham, MA USA), and processed using a Geno/Grinder^®^ 2010 (SPEX^®^ SamplePrep LLC, Metuchen, NJ, USA). Fecal swabs and intestinal tissue homogenate were diluted in 1 mL of phosphate-buffered saline solution (PBS). RT-qPCR conditions and methodology have been described previously [12]. Briefly, nucleic acids were extracted from 100 µL sample aliquots using MagMAX™ Pathogen RNA/DNA Kit (Thermo Fisher Scientific, Waltham, MA, USA) with a KingFisher Flex Purification System (Thermo Fisher Scientific, Waltham, MA, USA) following the instructions of the manufacturer. Primer pairs were designed to amplify overlapping amplicons targeting the complete genomes of two previously published PoAstV3 sequences (Table 1, GenBank accession Nos. JX556691 and KY940545). The viral genomic RNA was amplified using a 25 µL reaction utilizing custom qScript^®^ XLT One-Step RT-PCR Kit (Quanta Biosciences, Gaithersburg, MD, USA) following the manufacturer’s recommendations. Each primer was present in the final reaction at 320 nM, and 4 µL of RNA template was used per reaction. PoAstV3 RNA amplification was performed on an Applied Biosystems SimpliAmp thermal cycler (Thermo Fisher Scientific, Waltham, MA, USA) under the following conditions: initial reverse transcription at 48 °C for 20 min, followed by initial denaturation at 94 °C for 3 min and 45 cycles of denaturation at 94 °C for 30 s; annealing at 50 °C for 50 s; extension at 68 °C for 90 s; and final elongation at 68 °C for 7 min. Phosphate buffered saline was extracted as a negative extraction control and nuclease-free water was used as a negative amplification control. The RT-qPCR products were visualized using QIAxcel Advanced System (Qiagen, Germantown, MD, USA) and purified using the ExoSAP-IT PCR Product Cleanup Reagent (Thermo Fisher Scientific, Waltham, MA, USA) according to the manufacturer’s instructions. Sequencing was completed via Sanger sequencing with the BigDye™ Terminator v3.1 cycle sequencing kit (Thermo Fisher Scientific, Waltham, MA, USA) on a 3730xl DNA Analyzer (Thermo Fisher Scientific, Waltham, MA, USA) at the Iowa State University DNA Facility. Sequences were assembled using Geneious Prime 2020.2.4 software.

### 2.3. Phylogenic and Sequence Analysis

PoAstV3 sequences obtained were aligned using ClustalO webserver under default settings with 47 full genome neurotropic *mamastrovirus* strains retrieved from GenBank. Phylogenetic trees were generated using the neighbor-joining algorithm with MEGAX and rendered with interactive Tree Of Life (iTOL) [41,42,43,44]. In addition to the PoAstV3 sequences obtained in this study, 14 more PoAstV3 strains from the USA (GenBank accession number: JX556691, KY940545, MT394895, MT394896), Europe (GenBank accession number: MK962341, MK962342, KY073229, KY073231, KY073232), and Japan (GenBank accession number: LC201595, LC201596, LC201597, LC201598, LC20159) were retrieved to compare intrinsic PoAstV3 features. *Mamastrovirus* 3 GER/L00919-K17/2014 (LT898424), which was previously identified in other studies to cluster closely to PoAstV3 strains was also included in the analysis [45,46]. All sequences were retrieved from GenBank. In addition, PoAstV3 ORF1a, ORF1ab, and ORF2 were identified and translated using Jalview 2.11.1.3 and further aligned with ClustalO webserver under default settings to obtain nucleotide (nt) and amino acid (aa) percent identity matrices [41,47].

### 2.4. UTRs and Frameshift Signal Analysis

The 5′UTR (from the conserved pentamer CCAAA up to the first initiation codon), frameshift region (from the conserved shift heptamer sequence AAAAAAC to the first upstream UGA termination codon), and the stem-loop 2 motif (s2m) plus the 3′UTR (i.e., region upstream from the conserved sequence CGAGGCC including the ORF2 termination codon) of PoAstV3 strains were identified, trimmed, and realigned with ClustalO webserver under default settings using Jalview 2.11.1.3. Sequences with incomplete 5′ and 3′UTR were excluded from the analysis. Additionally, a 31 nt poly (A) tail, as the one present on PoAstV3 USA/IA/7023/18 (GenBank accession number: KY940545), was added to all PoAstV3 3′UTRs. The consensus sequence of both 5′UTR and frameshift signal regions were modeled using RNAfold webserver under default settings except for temperature rescaling at 39 °C to represent physiologic normal parameters of swine [48,49]. Previously described sequence frameshift signal of human astrovirus 2 (HAstV-2, GenBank accession: L13745) was modeled as comparison [33]. The s2m plus 3′UTR regions of PoAstV3 were segregated upon what country they were originally identified (i.e., USA, Japan, Hungary, Spain, and Germany) and the resulting consensus sequences for each geographic cluster were modeled using RNAfold webserver under default settings with temperature rescaling to 39 °C. Additionally, the s2m plus 3′UTR consensus sequences were analyzed for the presence of pseudoknotted structures with nested pseudoknots using IPknot webserver using the McCaskill model with refining parameters and weight of base pairs set at 50 in all levels [50]. Maximum expected accuracy (MEA) structures were visualized using FORNA webserver [51]. RNA conserved motifs for putative binding sites previously identified in human astroviruses at their 5′UTR for SRSF2 (GGCCUUUG), SRSF5 (ACAGG, CAAAAG), SRSF3 (UCUAC), SRSF6 (UGUAUA), hnRNPE2 (UUAU), TIA1 (AUUUUCU), and PTB (UUCU, UUCUCU) and their 3′UTR for SRSF2 (UGUCUCUG), SRSF3 (CUCUGUU), SRSF5 (UCAGA), SRSF6 (UACAGC), hnRNPE2 (UUAU/UUAG), TIA1 (UUAUUUU) and PTB (UCUU) were identified in PoAstV3 strains using find function with Jalview 2.11.1.3 [36,47].

### 2.5. Identification of Putative Genome-Linked Virus Protein (VPg)

The presence of a putative VPg previously identified between residues 664 and 758 (human) and residues 666 to 752 (mink) of ORF1a was evaluated [52,53]. Predicted N-terminal proteolytic cleavage site [Q(K/A)] located upstream of conserved motif KGK(N/T)K and C-terminal proteolytic cleavage site [Q(P/A/S/L)] downstream of C- terminal EEY-like motif were identified within the ORF1a of USA consensus sequence and its molecular weight was calculated using Expasy Compute pI/Mw tool [54]. USA nsp1a consensus sequence was further analyzed with DisoRDPbind webserver to identify putative disordered RNA-protein binding regions [55].

### 2.6. Linear Antigen Epitope Prediction

The consensus capsid protein sequence of USA, Japanese, Hungarian, and Spanish PoAstV3 strains and the capsid protein sequence of German strain GER/L00919-K17/2014 were analyzed under default settings with SVMTriP webserver and the Immunomedicine group’s predicted antigenic peptides web-based tools for the prediction of linear epitopes [56,57]. Recommended epitopes predicted with SVMTriP webserver (score ≥ 0.85), and sequence motifs predicted by the Immunomedicine group’s web-based tool (antigenic propensity ≥1.2) were identified and further analyzed using VaxiJen v2.0 webserver with a threshold value of 0.4 [58]. Probable antigens identified in USA capsid protein with VaxiJen v2.0 webserver, were further identified in individual USA strains using find function with Jalview 2.11.1.3 and the aa % identity of these regions were evaluated for point mutations.

## 3. Results

### 3.1. Diagnostic Samples

Sixty-four samples (CNS tissue (*n* = 61), feces (*n* = 2), and intestinal tissue homogenate (*n* = 1)) from 32 diagnostic cases were available and retrieved. Twenty-nine of these cases (*n* = 29/32, 90.6%) had a diagnosis of polioencephalomyelitis due to PoAstV3 with CNS tissues available. In three cases (feces (*n* = 2) and intestinal tissue homogenate (*n* = 1)) PoAstV3 PCR-based detection was requested by the submitter. The age of affected animals in CNS cases ranged from 3-week-old pigs to adult sows. CNS cases by production stage were as follows: nursery (3 to 9 weeks of age (*n* = 19)), grow-finish (10 to 13 weeks of age (*n* = 4)), and adult (gilts and sows (*n* = 6)). Non-CNS samples consisted of feces from suckling piglets (5- and 8-day-old) and intestinal tissue homogenate from suckling piglets (7-day-old). All CNS cases were reported to originate in the state of Iowa, except for two cases that originated in Illinois. These cases occurred during January (*n* = 1), February (*n* = 4), April (*n* = 3), May (*n* = 2), June (*n* = 2), July (*n* = 6), August (*n* = 3), September (*n* = 3), October (*n* = 5) and December (*n* = 3). Cases were traced back to 18 unique sources (systems). Of the 32 diagnostic cases, twelve (37.5%) originated from unique, unrelated farms/systems, seven (21.8%) cases were identified to originate from a single farm/system, followed by three different farms/system with three (9.3%) cases each, and two different farms/systems with two (6.2%) cases each. Additional epidemiologic and clinicopathologic information for cases 3, 4, 7, and 8 had been published elsewhere [28,59]. A summary of metadata by case including farm and strain identification (ID), tissue sample, production category, state of origin, initial PoAstV3 RT-qPCR result, clinical signs of affected animals, and month of diagnosis are summarized in Table S1.

### 3.2. RT-qPCR and Sanger Sequencing

Twenty complete coding sequences (CDS) were obtained from the initial 64 samples retrieved (*n* = 20/64, 31.25%). The complete 5′UTR was obtained in all 20 coding sequences while the complete 3′UTR, excluding the poly (A) tail, was obtained in 19 of the coding sequences, in addition to a partial 5′ termini (42 nt) of the 3′UTR of strain USA/IA/38290C/20. These 20 sequences originated from 16 different cases (*n* = 16/32, 50%) representing 11 unique sources (systems), with the most sequences (*n* = 19/20, 95%) obtained from CNS cases and only one sequence from feces (Table S1). Genome sequences from strains with a complete 3′UTR (*n* =19) ranged from 6328 to 6434 nt in length with a GC content of 46.7% to 47.7% (Table S2). Sequences were deposited at GenBank under accession numbers MW653747-MW653753 and MW732145-MW732157.

### 3.3. Phylogenic and Sequence Analysis

**Figure 1:**
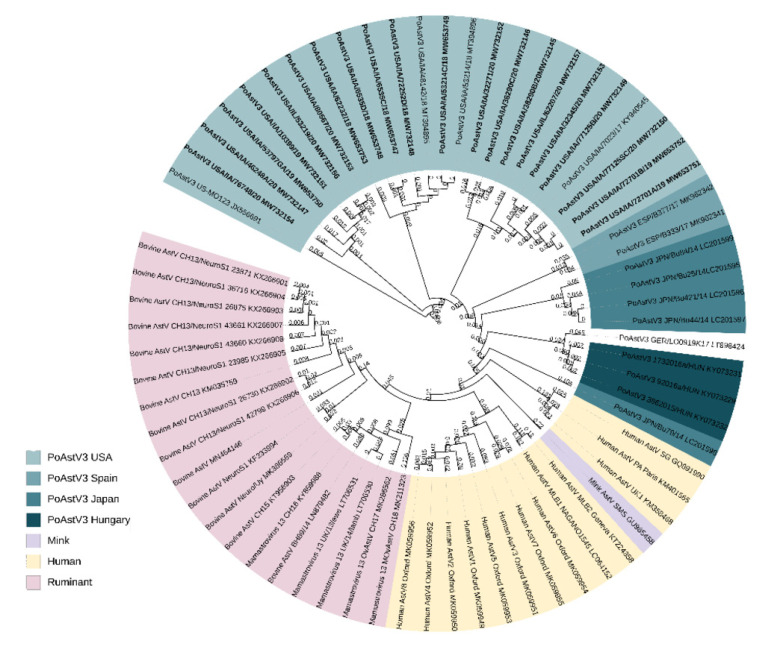
Phylogenic tree of selected full-genome neurotropic *Mamastrovirus* strains and 20 novel full-genome PoAstV3 strains identified in this study (bold). All novel PoAstV3 strains identified in this study clustered with PoAstV3 strains previously described. Nucleotide sequences were aligned using ClustalO webserver under default settings. The phylogenic tree was generated using the neighbor-joining algorithm using MEGAX and rendered with iTOL.

All PoAstV3 strains obtained from diagnostic cases clustered with other PoAstV3 strains previously described in the USA and other neurotropic strains identified to belong to the VA/HMO phylogenetic clade within MAstV genogroup II (Figure 1). PoAstV3 sequences clustered in three distinct clusters within USA strains. Nine of these novel strains (GenBank accession: MW732152, MW732145, MW732146, MW732157, MW732155, MW732179, MW735150, MW653752, MW653752) clustered with previously described neurotropic PoAstV3 strain USA/IA/7023/2017 (KY940545); six novel strains (GenBank accession: MW653753, MW732156, MW732151, MW653750, MW732154, MW732147) clustered with original fecal strain US-MO 123 (JX556691); and five novel strains (GenBank accession: MW732153, MW653747, MW653748, MW653749, MW732148) clustered with neurotropic strains USA/IA/48142/2018 (MT394895) and USA/IA/53214/2018 (MT394896).

The ORF1a, ORF1ab, and ORF2 of USA strains are composed of 855, 1356, and 809 residues, respectively. The nt percent identity of USA strains varies from 89.74–100%, 91.09–100%, and 90.71–100% for ORF1a, ORF1ab, and ORF2, respectively. The aa percent identity of USA strains vary from 95.26–100%, 96.3–100%, and 93.51–100% for nsp1a, nsp1ab, and capsid protein, respectively (Table 2). Among all PoAstV3, the lowest nt/aa percent identity for ORF1a/nsp1a, ORF1ab/nsp1b and ORF2/capsid protein was 84.26/92.04, 85.94/93.26 and 64.44/64.05, respectively (Tables S3–S5). Among systems with multiple cases the sequence homology was the following: Farm/system B (*n* = 4), ORF1a (99.68–99.96%), nsp1a (99.53–100%), ORF1ab (99.68–99.9%), nsp1ab (99.63–99.93%), ORF2 (99.76–99.92%), capsid protein (99.76–99.88%); Farm/system N (*n* = 3) ORF1a (99.68–99.96%), nsp1a (96.21–99.88%), ORF1ab (91.33–99.19%), nsp1b (97.04–99.85%), ORF2 (91.93%), capsid protein (94.74–100%); Farm/system C (*n* = 2) ORF1a (90.65%), nsp1a (95.73%), ORF1ab (91.82%), nsp1b (96.45%), ORF2 (92.18%), capsid protein (94.61%) (Tables S3–S5).

### 3.4. UTR Analysis

#### 3.4.1. 5′UTR Analysis

**Figure 2:**
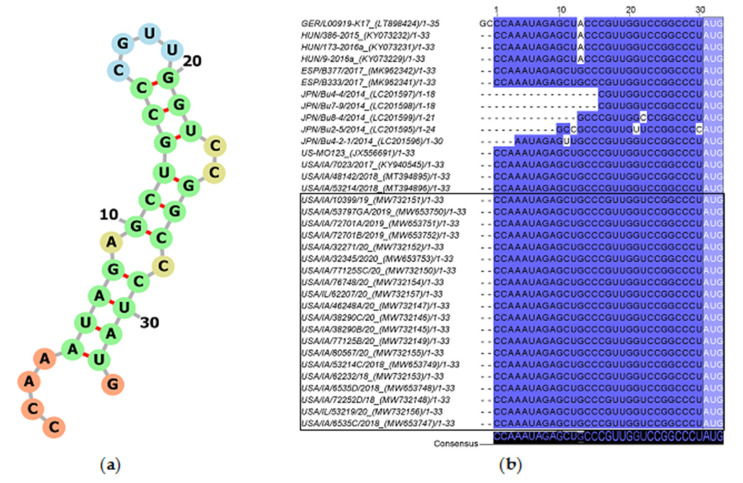
Consensus 5′UTR secondary RNA structure of USA PoAstV3 (**a**). Percent identity of 34 PoAstV3 5′UTR and 5′UTR of strain GER/L00919-K17/2014 (**b**). Darker blue tone indicates homologous bases. Note the inclusion of 5′UTR of strain GER/L00919-K17/2014 with initial 5′ GC bases only present in this strain. GenBank accession within parentheses. The start codon is included on both secondary structure (**a**) and percent identity plot (light blue box). Sequences identified in this study are denoted by a black box.

The 5′UTR of USA, Spanish, and Hungarian PoAstV3 strains is 30 nt long while German strain GER/L00919-K17/2014 is 32 nt in length given a GC insertion at the 5′ termini. The 5′UTR of USA and Spanish PoAstV3 strains are identical and differ from Hungarian strains and German strain GER/L00919-K17/2014 by a single mutation (G>A). The Hungarian strains and German strain GER/L00919-K17/2014 are identical if initial GC insertion is absent from strain GER/L00919-K17/2014. The 5′UTR of all Japanese strains appears incomplete although their 3′ termini (CCGGCCCU) are identical to other PoAstV3 strains, except for JPN/Bu-5/2014 (Figure 2). The 5′UTR consensus sequence folds into a stem with an internal loop (A, C), a loop (CGUU), and a bulge (CC) and has a free energy score of −7.80 kcal/mol. (Figure 2). A suboptimal Kozak consensus sequence ACCAUGG (initiation codon underlined) is found as part of the UTR at the 3′ termini of the stem including the ORF1a/1ab initiation codon. The minimum free energy (MFE) of the optimal secondary structure of consensus 5′UTR was −7.06 kcal/mol.

#### 3.4.2. Frameshift Analysis

**Figure 3:**
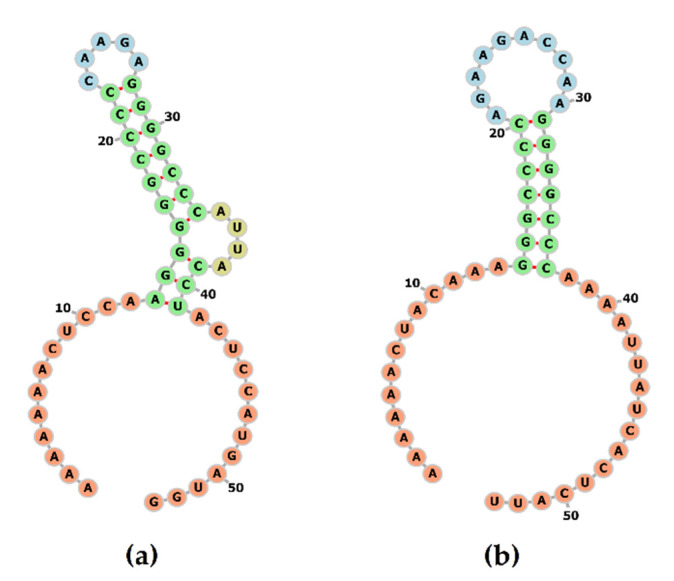
Secondary structure of the frameshift signal of PoAstV3 (**a**). The structure is composed of a stem motif with a conserved loop and bulge. The secondary structure of the frameshift signal of human astrovirus 2 (GenBank accession L13745) was modeled for comparison (**b**).

The conserved shift heptamer sequence (AAAAAAC) is located consistently in all PoAstV3 strains at position 2516–2523, with a conserved UGA stop codon located 39 nt upstream of this sequence motif. A repeated, *in tandem*, AUG codon is located in a −1 position from the termination codon, and a single nucleotide substitution (2555 C > U) is observed in strains USA/IA/76780/20 and JPN/Bu2-5/2014 in contrast to all other strains. The secondary RNA structure of this conserved region is characterized by the presence of a loop motif (CAAGA) and a bulge (AUUA) (Figure 3a). The predicted optimal MFE of this structure was −16.93 kcal/mol.

#### 3.4.3. 3′UTR Analysis

**Figure 4:**
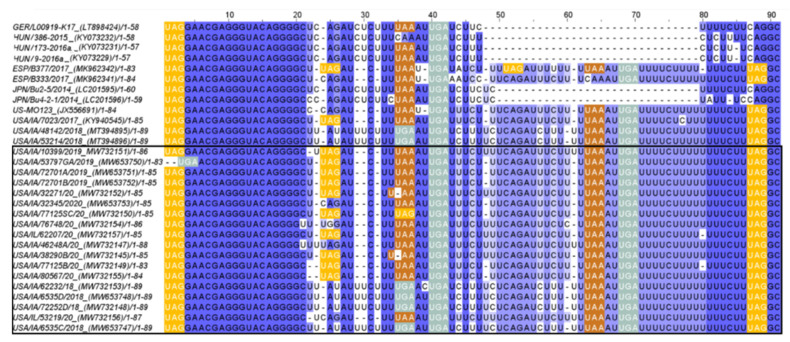
Sequence alignment of the 3′UTR region of PoAstV3 strains. Sequences with an incomplete 3′UTR region were omitted. Note insertion-deletion (IN/DEL) region present between bases 48–79 in USA and Spanish sequences and absent in Hungarian and Japanese strains, and GER/L00919-K17/2014. Stop codons UAG, UAA and UGA, in amber, ochre, and opal boxes, respectively. Dark blue indicates higher sequence percent identity. Poly (a) tail regions were excluded. Sequences were aligned using ClustalO webserver under default settings. Sequences identified in this study are denoted by a black box.

The length of the 3′UTR of PoAstV3 without the poly (A) tail, varies between 54 nt in Hungarian strains (KY073229, KY073231) to 86 nt in length in USA strains (MT394895, MT394896, MW653747, MW653748, MW732148, MW732153). The 3′UTR of USA strains varies between 80 nt to 86 nt in length. The 3′UTR of GER/L00919-K17/2014, Japanese (LC201595, LC201596), and Spanish strains (MK962341, MK962341) are 58 nt, 59–60 nt, and 83–84 nt in length, respectively. In all strains, the canonical stop codon UAG is consistently located 15 nt upstream of the start of the s2m except for strain USA/IA/53797GA/2019 (MW653750) that possess a UGA (opal) stop codon 16 nt upstream from the start of s2m. All PoAstV3 strains possess multiple stop codons within their 3′UTR (Figure 4).

**Figure 5:**
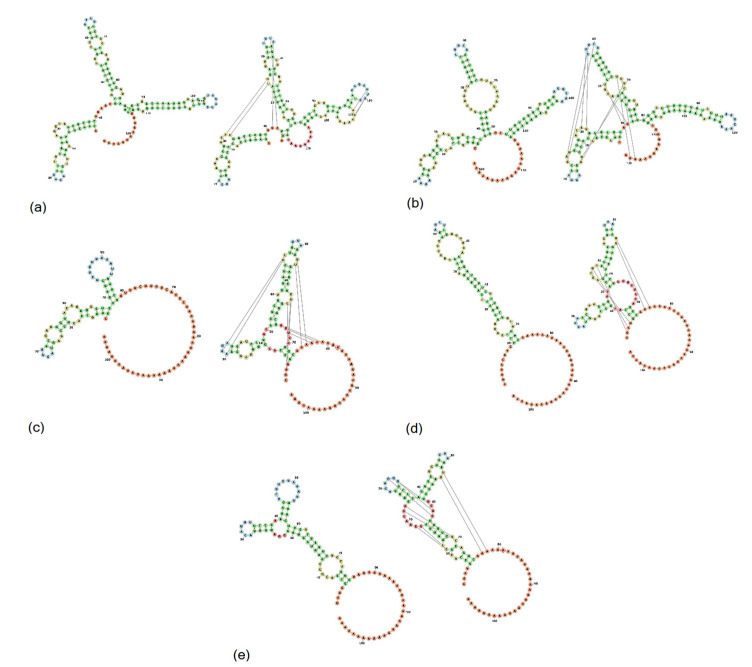
Secondary structure of the consensus PoAstV3 3‘UTR region of USA (**a**), Spanish (**b**), Hungarian (**c**) strains, strain GER/L00919-K17/2014 (**d**) and consensus Japanese strain (**e**). Secondary structures of (**a**–**e**) are illustrated without (**left**) and with pseudoknotted interaction (**right**). Green, yellow and blue bases indicate stems, interior loops, and hairpins, respectively. Red and orange bases indicate multiloops, and 5′ and 3′ unpaired regions, respectively.

In USA and Spanish strains, a net insertion of up to 32 nt in their 3′UTR causes the extension of the 3′UTR in contrast with Hungarian and Japanese strains, and GER/L00919-K17/2014. Secondary RNA structures similar to the s2m are predicted in USA, Hungarian, and Spanish 3′UTR consensus region but are not evident in Japanese 3′UTR consensus and GER/L00919-K17/2014. In the 3′UTR consensus region of USA and Spanish strains, this motif is followed by two stem structures with multiple internal loops. The 3′UTR consensus region of Hungarian PoAstV3 has only a single stem structure with a prominent loop. In contrast, the secondary 3′UTR structure of the Japanese consensus strain and GER/L00919-K17/2014 do not form a classical s2m structure and instead form a single stem with an internal loop, a bulge and a multi-branched loop, and a stem-loop with internal loops, a bulge, and a hairpin loop, respectively. Distant base-pairing regions and pseudoknots were further detected with IPknot webserver. Multiple base-pairing regions were predicted between the s2m and adjacent stem-loop motif within the 3′UTR of USA and Spanish consensus sequences. The secondary structure of the Hungarian 3′UTR consensus region initially modeled with RNAfold, transformed into a single multi-branched stem-loop similar to the multibranched predicted stem-loop of GER/L00919-K17/2014. Additional base-pairing regions were predicted within the multi-branched structure initially modeled for Japanese 3′UTR region, and between this structure and the poly (a) tail (Figure 5). The MFE of optimal secondary structures of USA and Spanish 3′UTR was −16.07 and −16.27 kcal/mol, respectively. The MFE of optimal secondary structures of Hungarian and Japanese strains, and strain GER/L00919-K17/2014 3′UTRs was −13.22, −17.06, and −14.25 kcal/mol, respectively.

**Figure 6:**
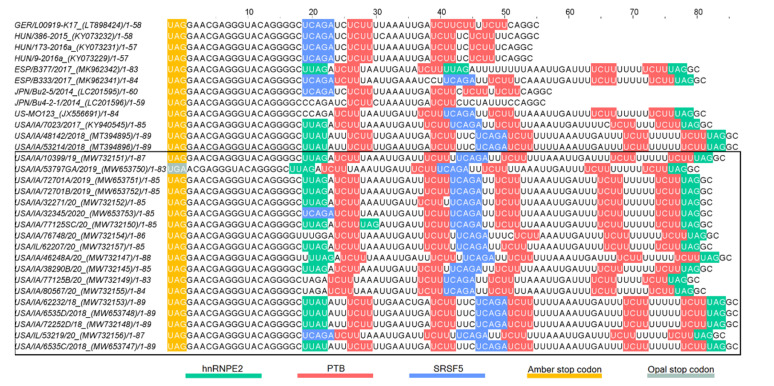
Putative protein binding sites of the 3′UTR of PoAstV3. RNA binding sites for serine/arginine-rich splicing factors 5 (SRSF5 [UCAGA, blue bases]), heterogeneous nuclear ribonucleoprotein E2 (hnRNPE2 [UUAG/UUAU, green bases]) and polypyrimidine tract-binding protein (PTB [UCUU, red bases]). UAG and UGA stop codons in amber and opal boxes, respectively. Sequences identified in this study are denoted by a black box.

**Figure 7:**
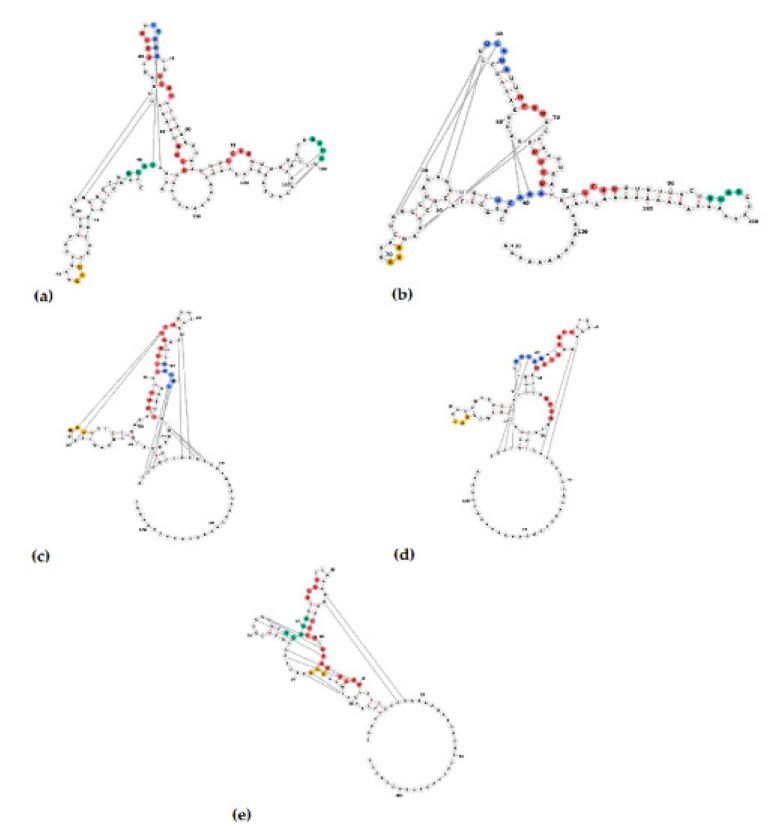
Secondary structure of 3′UTR of PoAstV3 and RNA binding sites for serine/arginine-rich splicing factor 5 (SRSF5 [UCAGA, green bases]), heterogeneous nuclear ribonucleoprotein E2 (hnRNPE2 [UUAG/UUAU, blue bases]) and polypyrimidine tract-binding protein (PTB [UCUU, red bases]). USA consensus sequence (**a**), strain ESP/B333/2017 (**b**), Hungarian consensus sequence (**c**), GER/L00919-K17/2014 (**d**) and JPN/Bu2-5/2014 (**e**). UAG stop codon (amber bases).

Multiple putative protein binding sites were identified in the 3′UTR of PoAstV3. Putative binding sites for PTB were consistently found in all sequences, ranging from two motifs (in JPN/Bu4-2-1) up to four intercalated motifs in USA strains. In Hungarian and Japanese strains, as in strain GER/L00919-K17/2014, only putative binding sites for PTB and hnRNPE2 were identified. In all other PoAstV3 strains at least one motif for PTB, hnRNPE2 and SRSF5 were identified (Figure 6 and Figure 7). No putative protein binding sites were identified on the 5′UTR of PoAstV3.

### 3.5. Identification of Putative VPg and RNA-Protein Binding Regions

VPg consensus protein sequence [QAKGKNKKHHRRRGGKKRRAVWSEEEYKELLEKGFSKSQL] located within residues 640 to 679 of USA strains and with a molecular weight of approximately 4.81 kDa was identified between putative N-terminal proteolytic cleavage site [Q(K/A)] and a C-terminal proteolytic cleavage site [Q(P/A/S/L)]. A majority of USA strains from this study as well as those already in GenBank have an arginine residue at position 657 except for USA/IA/77125B/20 (MW732149), USA/IA/80567/20 (MW732155), USA/IA/7023/2017 (KY940545), USA/IA/77125SC/20 (MW732150), USA/IA/72701A/2019 (MW653751), and USA/IA/72701B/2019 (MW653752) which have a histidine at this site. The VPg consensus sequence is identical in all other PoAstV3 sequences except for strains GER/L00919-K17/2014 (648:H>Y), JPN/Bu8-4/2014 (647: K>N; 655: K>R), and Hungarian strain (648: H>N) (Figure S1). Three RNA-protein binding regions were identified within the nsp1a with DisoRDPbind webserver. Putative RNA-protein binding regions were located within residues 316-392 ([AAFTLSAGVFMPDVRYSDLVRGQLVVFLVLVFNYLVVMMSLPNWLVFSLVVGYRVLRVLTFLVAEKVEVRGPDGKV]), 633- 647 ([SATTFIQAKGKNKK]) and residues 807-813(MLMWEK). Residue RNA-binding propensity scores are presented in Supplemental Material 1.

### 3.6. Linear Antigen Epitope Prediction

Results, sequences, and scores of predicted linear epitopes and antigens are summarized in Table 3. SVMTriP online tool predicted four recommended linear epitopes in strain GER/L00919-K17/2014 and three linear epitopes for Hungarian, Spanish, and Japanese consensus sequences, respectively. Two recommended linear epitopes were identified in the USA consensus sequence. The Immunomedicine antigenic peptides web-based tool predicted three linear epitopes for Spanish consensus sequence, two linear epitopes in the USA, Hungarian, and Japanese consensus sequences, and one linear epitope for GER/L00919-K17/2014. Of the fifteen epitopes predicted by SVMTriP online prediction tool and the eight epitopes predicted by Immunomedicine antigenic peptides web-based tool, VaxiJen v2.0 prediction tool corroborated the probable antigenicity of ten and four linear epitopes, respectively. Epitopes predicted by both SVMTriP and VaxiJen v2.0 online prediction tools included [TNCADVLAYKHEKWGDNLKF] identified in all consensus strains and GER/L00919-K17/2014 with the exception of the USA consensus strain and [AKHMSTAYEKLPLNALTVGE] identified in all consensus sequences and GER/L00919-K17/2014 except for the Japanese consensus strain. Epitopes predicted by both Immunomedicine antigenic peptides and VaxiJen v2.0 online prediction tools included [GNFYCLVSAPVISNVDQAPVTIVF(D/N)KVATPKLSTSSSLSHMRVSVSSVQPGTYGVIYT] in USA and Spain consensus sequences, [GNFYCLVSAPIISNVDQAPVTIVANKVATPKLSTSSSLSHMRVSVSSVQPGTYGVIYT] in Japanese consensus sequence, and [GNFYCLVSAPVVSDVNQAPVTIVSNKVATPKLSTSSSLSHMRVSVSSVQPGMYGVIYTLGS] in GER/L00919-K17/2014. Within USA PoAstV3, potential antigens [AKHMSTAYEKLPLNALTVGE], [YKHEKWGDNLKFSSWLVRFT] and [GNFYCLVSAPVISNVNQAPVTIVFNKVATPKLSTSSSLSHMRVSVSSVQPGTYGVIYT] were found without any mutation in seventeen, twelve and two strains, respectively.

**Figure 8:**
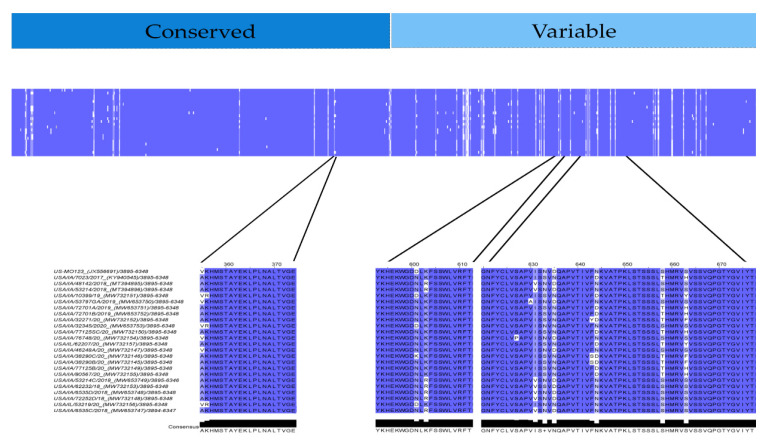
Capsid protein percent identity plot of USA PoAstV3 from this study. Blue and white areas indicate areas of higher and lower homology, respectively. Amino acid homology of linear epitopes and probable antigens of USA PoAstV3 strains identified in this study.

Point mutations in [AKHMSTAYEKLPLNALTVGE] were located at position 1 in four strains ([V>A] GenBank accession: JX556691, MW732147, MW732150, MW732154) and in positions 1–2 in three strains ([VR/AK] GenBank accession MW732151, MW653751, MW732156) (Figure 8). Point mutations in [YKHEKWGDNLKFSSWLVRFT] were located at position 9 in four strains ([D/K>N] GenBank accession: JX556691, MW732151, MW653753, MW732146, MW732156) and in position 11 in seven strains ([K/R] GenBank accession MT394895, MT394896, MW653748, MW653749, MW732153, MW653748, MW732147). Potential antigen [GNFYCLVSAPVISNVDQAPVTIVFDKVATPKLSTSSSLSHMRVSVSSVQPGTYGVIYT] had low conservation at positions 12,14,16,25, 39 and 44. Additionally, single mutations differing from the consensus were found in strain MW732145, MW732146 and MW732152 (position 24 [F>S/Y]), strain MW653750 (position 11 [V>A]) and strain MW732154 (position 8 [S>P]) (Figure 8).

## 4. Discussion

This study characterizes the CDS, 3′ and 5′UTRs of the largest collection of PoAstV3 sequences (*n* = 20) associated with polioencephalomyelitis in swine originating from 11 individual farms/systems. Consistent with other reports, PoAstV3-polioencephalomyelitis affects swine at different stages of production (weaning, grow-finish, adult animals) [15,42]. Additionally, a clear association with winter months and the occurrence of PoAstV3 neurotropic infections, as other authors have previously suggested, has not been corroborated in this study [15]. On the contrary, PoAstV3 CNS cases did not have a clear seasonal distribution: summer (*n* = 9), autumn (*n* = 8), and winter (*n* = 8), and spring (*n* = 4). This may be a result from current intensive production practices or indicate that seasonal distribution is not a prominent feature of PoAstV3 ecology.

As with other astroviruses, the genome of PoAstV3 contains three ORFs. PoAstV3 USA strains identified in this study follow the ORFs delimitation described for sui generis US-MO 123 strain with a nsp1a, nsp1ab and capsid protein composed by 855, 1356 and 809 residues, respectively. The range sequence similarity of nsp1a, nsp1ab and the capsid protein of the USA strains characterized in this study is 94.79–100%, 96.3–100% and 93.51–100%, respectively. Not surprisingly, the lowest percent homology was observed in the capsid protein. These twenty novel USA strains cluster with previously described strains into three well-defined clusters. Nine strains originating from six farms/systems and six strains originating from four farms/systems clustered with the original neurotropic PoAstV3 strain USA/IA/7023/2017 (KY940545) and original fecal strain US-MO 123 (JX556691), respectively. Five of these novel strains originating from 2 farms/systems clustered with strains USA/IA/48142/2018 (MT394895) and USA/IA/53214/2018 (MT394896) which recently resulted in PoAstV3-polioencephalitis following experimental inoculation of CDCD pigs [12,21,28,31,60,61]. Interestingly, strains from one farm/system (Farm N) clustered with both strain USA/IA/7023/2017 (KY940545) and strain US-MO 123 (JX556691). Based on the genetic analysis of strains originating from system N, it appears that within a system distinct PoAstV3 strains can cause neurological disease in sows with one strain also been found in feces of suckling piglets (Table S1). In contrast, on Farm B a subset of highly homologous PoAstV3 strains (>99% nt ORF2) was detected in multiple polioencephalomyelitis cases spanning a year (2018). Similarly, on Farm E and O highly homologous PoAstV3 strains (>99% nt ORF2) were detected in two individual animals experiencing PoAstV3-associated neurological disease at the same time.

Previous studies involving neurotropic mamastroviruses have reported unsuccessful attempts at viral isolation. We have also pursued virus isolation in a few of the samples identified in this study using multiple cell lines (i.e., PK-15, ST, BHK, Vero cells [data not shown]) with similar negative results. The inability to isolate neurotropic strains from clinical samples appears to be a common factor for many neurotropic astroviruses and this impediment may be partially compensated by the development of robust reverse genetics systems, as previously described, or by the development of specific permissive cell culture systems [44]. Hence, the study and characterization of RNA motifs associated with virus replication and translation initiation, as those commonly found in both UTRs may serve as an initial approach to develop future studies [17,38,40,62].

The 5′UTR of PoAstV3 is highly conserved among all strains. A single point mutation is observed in Hungarian strains in comparison with USA and Spanish strains. This point mutation is located at position 13 involving a G>A substitution. Previously, the motifs CCAA, UGGU and GGCC were identified in human astrovirus and are present in all PoAstV3 strains located at position 1, 19 and 25, respectively [36,62]. Strain GER/L00919-K17/2014 has nucleotides GC as initial sequence bases, extending its UTR up to 32 nt in contrast to the 5′UTR of PoAstV3, although if absent, the remaining of the 5′UTR is identical to Hungarian strains. The 5′UTR of all Japanese strains appears incomplete due to the absence of the pentamer CCAAA, although available 5′UTR segments have a high degree of homology with other PoAstV3, and they all possess the invariable motif GGCC. This high degree of sequence conservation within the 5′UTR of PoAstV3 and other mamastroviruses, and the presence of conserved motifs among PoAstV3 and human astroviruses supports the notion of common ancestral origins and possible recombination events between members of the genus *Mamastrovirus*, including human astroviruses.

In single-stranded RNA viruses, including human astroviruses, viral translation is initiated at the 5′UTR and previous studies modelling the 5′UTR of human astrovirus have shown a high sequence-based, secondary RNA structural degree of conservation [36,62,63,64]. Our results indicate that the short 5′UTR sequence of PoAstV3, and therefore its secondary RNA structure, is highly conserved in strains found in different continents. Furthermore, our analysis suggests that the high degree of conservation of the 5′UTR of PoAstV3 is highly regulated and may possess an essential role in the virus life cycle. In human strains, the 5′UTR has been shown to contain putative protein binding sites; although, our analysis indicate there is not marked homology within predicted binding site motifs (e.g., PTB, SRSF2, SRSF5 and SRSF6) found in humans astrovirus and the 5′UTR of PoAstV3. Thus, this may suggest a plausible explanation of why PoAstV3 have not been successfully isolated in cell culture systems using human cell lines [16,17,22].

In members of the genus *Mamastrovirus*, experimental evidence has previously inferred the presence of a VPg linked to infectious viral RNA, and in human astrovirus, this has been shown to be essential for in vitro infectivity [36,45,46]. In this study we identified a conserved polyprotein within the ORF1ab of USA PoAstV3 strains spanning between previously described cleavage sites encompassing the putative human astrovirus VPg. Furthermore, we found the RNA binding site [SATTFIQAKGKNKK] between residues 633-647 containing the conserved motif KGK(N/T)K previously characterized as the N-terminal end of calicivirus VPg, and the conserved proteolytic cleavage site [Q(K/A)] previously described in human astroviruses. In addition, the conserved motif EEY found in calicivirus VPg (including a tyrosine residue postulated to covalently associate with viral RNA) was also identified. This suggests that both KGK(N/T)K and EEY motifs of PoAstV3 putative VPg could bind viral RNA. Interestingly, the molecular weight of the putative PoAstV3 VPg is approximately 4.81 kDa, considerably smaller than those found in human astrovirus and caliciviruses with an estimate molecular weight of 11 kDa and 13-15 kDa, respectively [36,45,47,48]. Future research directed to infer the role of this putative PoAstV3 VPg can aid in the development of in vitro infectivity studies designed to assess the relevance of this protein in cell culture systems [49,50].

In contrast to the 5′UTR, multiple protein binding sites analogous to human astroviruses are observed in the 3′UTR of PoAstV3. The 3′UTR secondary structure of PoAstV3s is complex and may adopt multiple secondary RNA conformations. This appears to be related to the presence of the duplicated tandem motif UUGAUUUUCU(U/C)UUUUUUCUUUAGGC found in USA and Spanish strains (Figure 3). This sequence motif is absent in other European and Japanese strains and clearly demarks the conformation of the secondary structure and putative binding sites of PoAstV3 strains. The 3′UTR of Hungarian and Japanese PoAstV3 strains and German strain GER/L00919-K17/2014 is shorter and therefore possess fewer protein binding sites in comparison to USA and Spanish strains (Figure 5). In GER/L00919-K17/2014, ESP/B333/2017, USA/IL/53219/20 and all Hungarian strains, SRSF5 is the first binding motif found at position 19–23 after the ORF2 stop codon. This binding site is replaced in 16 American strains and strain ESP/B377/2017 for hnRNPE2 binding site, spanning generally from nucleotides 19–22 upstream of the ORF2 stop codon. Adjacent to these motifs, the first PTB binding site is generally found spanning from nucleotides 24–29. Multiple intercalated PTB binding sites are found in all PoAstV3 strain. PTB motifs varies from two to four copies, with the fewest copies found in strain JPN/Bu4-2-1/2014 and the most in multiple USA strains. A dual binding site for PTB and hnRNEP2 is found as the last binding region of the 3′ termini in all USA and Spanish strains. In contrast to the 3′UTRs of human astroviruses, no putative binding sites for SRSF2, SRSF3 and SRSF6 were identified, indicating that RNA binding motifs for these cellular proteins are different from those identified for human astroviruses. These features and the absence of predicted RBPs in the 5′UTR of PoAstV3 could indicate that translation initiation and replication of PoAstV3 differs from that postulated for human astroviruses, and other cellular proteins and RNA binding sites may play a role in these processes. In addition, the 3′UTR of all PoAstV3 strains have multiple additional amber, ochre, and opal stop codons after standard amber stop codon (opal in strain USA/IA/53797GA/2019, Figure S2) suggesting the possibility of non-canonical elongation and termination mechanisms, and the use of stop-codon read-through strategy by PoAstV3, as it was previously described in other viruses [51,52]. Perhaps, the presence of repeat motif UUGAUUUUCU(U/C)UUUUUUCUUUAGGC containing multiple additional RBPs as observed in the 3′UTR of USA and Spanish strains is an evolutionary strategy used by PoAstV3 to maximize its replication by stop-codon read-through elongation and termination [53]. Future in vitro studies analyzing the interaction of swine orthologues for PTB, SRSF2, SRSFF3 SRSF5, SRSF6, TIA and hnRNPE2 with the 3′UTR of PoAstV3 may elucidate if binding of these cellular proteins is a requirement for PoAstV3 replication.

In seminal work, Willcocks and Carter described two distinct stem motifs within the 3′ termini of human astrovirus 1 (GenBank accession Z11682) [20,65]. In this study, two partially homologous regions are consistently found in the 3′UTR of PoAstV3 sequences. The first motif found in the 3′UTR of all PoAstV3 located immediately after the stop codon, GAACGAGGGUACAG, makes up the 3′ termini of s2m. The second motif, AAAUUGAUUU, is found consistently in USA and Spanish strains but not in other PoAstV3 strains and is part of a transition sequence region between subsequent stem structures found on these PoAstV3 strains. These two motifs [GA(A/T)CGAGGGUACAG, AAAUUGAUUU] are also conserved in the 3′UTR of all classical human genotypes (HAstV1 to HAstV8) as shown by Monceyron et al. constituting the s2m and s1m motifs, respectively; however, in PoAstV3 due to the differences in length of their 3′UTRs and secondary RNA structure, the second motif AAAUUGAUUU is predicted to participate in the folding of two stem structures instead of one (s1m) as seen in human astroviruses. This remarkable difference between human astroviruses, possessing two conserved stem motifs within their 3′UTR, and PoAstV3, that may present 1 to 3 stem structures in their 3′UTR, as shown by USA and Spanish strains (three stems), Hungarian strains (two stems) and Japanese strains and strain GER/L00919-K17/2014 (one stem) may indicate promiscuous binding by PoAstV3 strains with unknown cellular proteins (Figure 5). This difference is further accentuated when pseudoknots are predicted. Pseudoknot prediction indicates that the secondary structure of the 3′UTR of USA and Spanish strains remains as a three stem structure, while Hungarians strains adopt the conformation of a multibranched stem, similar to strain GER/L00919-K17/2014. Thus far, no predicted pseudoknots have been described in the 3′UTR of human astroviruses, although future in silico studies may corroborate the presence of pseudoknots in this region of human astroviruses and other members of the genus *Mamastrovirus*.

Recent work with human astroviruses has elucidated potential binding motifs for cellular proteins within their 3′UTR. Our analysis indicates that homologous binding regions are present in the 3′UTR of PoAstV3s. The elongation of the 3′UTR of USA and Spanish strains provides additional binding motifs when compared to other strains with shorter 3′UTRs (i.e., GER/L00919-K17/2014, Hungarian, and Japanese strains). The hairpin of the first and second stem motifs of USA and Spanish strains have binding sites for hnRNPE2 and SRSF5, respectively; and multiple PTB binding sites are predicted at the internal loops and bulges between these two stems. In contrast, GER/L00919-K17/2014 and Hungarian strains lack binding sites for hnRNPE2 within their secondary structure. Additionally, the stop codon is invariably present in the hairpin of stem loop analogous to s2m in all strains, with the exception of Japanese strains.

It is plausible that the null rate of success in isolating PoAstV3 in cell culture is related to intrinsic sequence motifs present in their genome as related to the secondary conformation adopted by RNA binding motifs. Future in vitro studies should contemplate the association of the cellular proteins described herein and focus on the identification of unknown cellular proteins relevant for the replications of PoAstV3 in cell culture systems. In addition, the increasing number of PoAstV3 genomes available in public databases can help to understand hidden complexities of this virus and aid in the development of permissible cell lines for neurotropic astroviruses; however, the validity of predicted RNA structures described in this study should be further correlated in context with the global RNA structure of PoAstV3. Future analysis considering the global structure of PoAstV3 and other neurotropic mamastroviruses would be valuable for assessing possible interactions of the UTRs with other genomic regions. Global analyses may elucidate secondary or tertiary structures potentially crucial for translation and replication of neurotropic mamastroviruses, and therefore, useful for the development of cellular culture systems to study viral pathogenesis.

In a previous study by Amimo et al. potential antigenic epitopes for PoAstV3 strain U460 were found in multiple locations of the ORF2. These antigenic epitopes were all located at the CP conserved region ([WRLTNLKIKCTPLVGPSAVTGSVYRVSLNLTQS], [MIEIHGLGKTSSTYKDEPWVGDLF], [PFQWLIKGGWWFVKKALGRSMNSDEVYYVYAS] and highly homologous consensus sequences [WRL**AD**LKI**R**CTPLVGPSAVTGSVYRLSLNLTQS], [M**V**EIHGLGKTSSTYKD**AD**WVGDLF], and [PFGWLIKGGWWFVKKA**I**GRS**NT**DE**T**YYVYAS] (mismatches in bold) were found in all USA PoAstV3 strains. Additionally, in our study, three antigenic epitopes were found at the CP of consensus USA PoAstV3 sequence. These epitopes both localized at the conserved ([AKHMSTAYEKLPLNALTVGE]) and variable regions ([YKHEKWGDNLKFSSWLVRFT], [GNFYCLVSAPVISNVNQAPVTIVFNKVATPKLSTSSSLSHMRVSVSSVQPGTYGVIYT]). Furthermore, two of our predicted antigenic epitopes ([YKHEKWGDNLKFSSWLVRFT], [GNFYCLVSAPVISNVNQAPVTIVFNKVATPKLSTSSSLSHMRVSVSSVQPGTYGVIYT]) are highly homologues to the 3′ termini of recombinant VP27 protein used in an indirect ELISA assay to detected IgA, IgG and IgM of CDCD pigs experimentally infected with PoAstV3 [31]. In astroviruses, VP27 is part of the dimeric spike protein and is considered highly variable and antigenic. Our results indicate that both antigenic epitopes identified are highly homologous within USA PoAstV3 strains and may elicit specific antibodies against PoAstV3. Future in vitro and in vivo studies are necessary to evaluate the efficacy and cross-protection of these antigens for the control of PoAstV3-asscociated CNS infections.

## 5. Conclusions

This study encompasses the analysis of the largest collection of PoAstV3-polioencephalomyelitis genomes, and the first analysis modelling the secondary structure of important putative regulatory RNA motifs in neurotropic *mamastrovirus*. Our results indicate contrasting differences to those previously described for human astrovirus, which may explain the inability of this virus, and perhaps other neurotropic mamastroviruses, to be isolated in cell culture. Finally, we described the presence of two highly conserved antigenic epitopes in the VP27 region of PoAstV3, which may allow the development of useful immunogens and diagnostic assays to diagnose and control cases of PoAstV3-associated polioencephalomyelitis.

## Figures and Tables

**Table 1:** List of forward and reverse primers used for sequencing of PoAstV3.

Primer ID	Sequence Target		Region Target
	POAstV3 US-MO123—JX556691 (5′-3′)	PoAstV3 USA/IA/7023/2017—KY940545 (5′-3′)	
F1_T7	ATGGTACCTAATACGACTCACTATAGCCAAATAGAGCTGCCCGTTGGTCC		ORF1
R1008	CAGAGTAGCGTACATCGGGCATG	CGGAGTAACGCACATCGGGCATG	ORF1a
F392	TCTCCCAAGAGCTCTCCCAA	NA	ORF1a
R1345	CCTGTTCCTGAGCCATCCTG	NA	ORF1a
F485	GCCGGTTAGGGTTCATCACA		ORF1a
R1591	GCAACTGACAGAGCACCTGA		ORF1a
F845	CATATTCAAAGGCCCAAGTTCTGGCCCTTG	CATACTCAAAGCCCAGGTTCTAGCTCTTG	ORF1a
R1662	CAGGTGAACCACTCATACCATTCCGA	CAGGTGAGCCACTCATACCATTCCGG	ORF1a
F1465	GTCCATCGTGTGCCGGATAAGGACATAG	GTCCACCGTGTGCCAGATAAGGACATAG	ORF1a
R2217	GTGTCTTGAGGCTCTTCCTTAACAGGC	GTGTCCTGAGGCTCTTCCTTAACAGGC	ORF1a
F2001	CCGTGCTGTCTGGTCAGAAGAAGAGTAC	CCGTGCTGTCTGGTCAGAAGAGGAATAC	ORF1a
R3203	CCACATTGGCCAGAGCGTGTC		ORF1a
F2462	GGGAGAAAGGCTTGGTTCCT		ORF1a
R3920	CAGGGAGGTTATGGCCAAGG		ORF2
F3127	GTTCTGACCCTATATTTGCACGTATAGGATGTCAC	GTTCTGACCCTATATTTGCACGTATAGGTTGTCAC	ORF1a
R4855	GTCACACGATCTAGTGTTCCAAGGGC	GTCACACGATCCAGAGTTCCAAGGGC	ORF2
F4308	CTTCTCAGTGCGAGTCTCGG		ORF2
R5326	AAAGTGGTGGCCGTAGATGG		ORF2
F4672	CCGCGACAGACGTGGTGG		ORF2
R5924	CGTCAGTCACATTATAGTTGCCAAGGG	CGTCAGCCACATTGTAGTTACCAAGGG	ORF2
F5486	GAGCTGGTAAAGGCATGGGT	NA	ORF2
R6283	TGGATAAGAGCGTCGGCATC		ORF2
F6108	CCGTCCGACCATGATTGACA		ORF2
R6352	CGAGGGTACAGGGGCTTAGA	TCTAAGCCCCTGTACCCTCG	3′ UTR
F5598	ACCTGGTATCGGCTGGACT		ORF2
R6405_M13	TGTAAAACGACGGCCAGTGCCTAAAGAAAAAAAAGAAAATCAATTTAA		3′ UTR
F2804	CCCTAATATAGCCGCCACGT	NA	ORF1a
R3987	GTGTCCTGATGTGCCTAGCA	NA	ORF2
F946	ACCCACAGTGAAAGGACAGC	NA	ORF1
F5623	CCTGGTATCGGCTGGACTGTT	NA	ORF2

Gray cells indicate identical primer sequence for both strains. PoAstV3 = porcine astrovirus type 3. ORF = open reading frame. UTR = untranslated region. NA = Not available.

**Table 2:** Range of nucleotide and amino acid percent identity of PoAstV3 strains.

Strain Origin	ORF1a	nsp1a	ORF1ab	nsp1ab	ORF2	Capsid Protein
USA	89.7–100%	94.7–100%	91.9–100%	96.3–100%	90.7–100%	93.5–100%
Japan	89.1–100%	95.2–99.9%	89.4–99.9%	95.3–99.9%	65.1–99.9%	65.6–99.9%
Spain	92.9%	96.3%	93.2%	96.6%	92.7%	95.6%
Hungary	99.6–99.7%	99.4–99.6%	99.6–99.6%	99.6–99.7%	99.1–99.4%	98.8–99.2%

Range of nucleotide and amino acid percent identity of PoAstV3 segregated by strain origin. Range values are indicated minimum-maximum percent identity. GER/L00919-K17/2014 is not included. ORF = open reading frame, nsp = non-structural protein.

**Table 3:** Linear epitope and antigen prediction results.

	Prediction Tool: SVMTriP Online Prediction Tool				
	Recommended epitopes	Score	ORF 2 region	ORF 2 position	VaxiJen v2.0 result (score)
USA	AKHMSTAYEKLPLNALTVGE	1	Conserved	355–374	Probable antigen (0.5478)
	YKHEKWGDNLKFSSWLVRFT	0.92	Variable	593–612	Probable antigen (0.8778)
Hungary	AKHMSTAYEKLPLNALTVGE	1	Conserved	292–311	Probable antigen (0.5478)
	TNCADVLAYKHEKWGDNLKF	0.953	Variable	522–541	Probable antigen (0.8269)
	LAKEVAKEVVKEEKRNQARS	0.893	Conserved	15–34	Probable non-antigen (0.1453)
Japan	IMTMTIPQRSNLARHMSTAY	1	Conserved	347–368	Probable non-antigen (0.0369)
	LAKEVAKEVVKEEKKVQARR	0.964	Conserved	77–96	Probable non-antigen (0.0094)
	TNCADVLAYKHEKWGDNLKF	0.892	Variable	591–610	Probable antigen (0.8269)
Spain	AKHMSTAYEKLPLNALTVGE	1	Conserved	355–374	Probable antigen (0.5478)
	TNCADVLAYKHEKWGDDLKF	0.879	Variable	585–604	Probable antigen (0.8161)
	ANQATSHSPVINNVFPVLGQ	0.862	Variable	496–515	Probable non-antigen (0.3368)
Germany	LAKEVAKEVVKEEKRFQARS	1	Conserved	77–96	Probable non-antigen (0.2543)
	PVFLGLNLGASSSTVDNIFL	0.999	Variable	544–563	Probable antigen (0.9103)
	AKHMSTAYEKLPLNALTVGE	0.911	Conserved	354–373	Probable antigen (0.5478)
	TNCADVLAYKHEKWGDNLKF	0.869	Variable	584–603	Probable antigen (0.8269)
	**Prediction tool: ** **Immunomedicine predicted antigenic peptides web-based tool**				
	Recommended epitopes	Score	ORF 2 region	ORF 2 position	VaxiJen v2.0 result (score)
USA	WVGDLFIVEVVG	>1.2	Conserved	298–309	Probable non-antigen (0.0845)
	GNFYCLVSAPVISNVNQAPVTIVFNKVATPKLSTSSSLSHMRVSVSSVQPGTYGVIYT	>1.2	Variable	620–677	Probable antigen (0.5617)
Hungary	WVGDLFIVEVVG	>1.2	Conserved	239–250	Probable non-antigen (0.0845)
	GNYYCLVSAPVISAVNQAPVTIVFDKVATPKLSTSSS	>1.2	Variable	561–597	Probable non-antigen (0.3268)
Japan	WVGDLFIVEVVG	>1.2	Conserved	308–319	Probable non-antigen (0.0845)
	GNFYCLVSAPIISNVDQAPVTIVANKVATPKLSTSSSLSHMRVSVSSVQPGTYGVIYT	>1.2	Variable	626–683	Probable antigen (0.5449)
Spain	GLWRLAGLKICCTPLVGPSAVTGSVYRLSLNL	>1.2	Conserved	195–226	Probable non-antigen (0.1431)
	WVGDLFIVEVVG	>1.2	Conserved	302–313	Probable non-antigen (0.0845)
	GNFYCLVSAPVISNVDQAPVTIVFDKVATPKLSTSSSLSHMRVSVSSVQPGTYGVIYT	>1.2	Variable	624–681	Probable antigen (0.5397)
Germany	GNFYCLVSAPVVSDVNQAPVTIVSNKVATPKLSTSSSLSHMRVSVSSVQPGMYGVIYTLGS	>1.2	Variable	619–679	Probable antigen (0.5507)

## Data Availability

The data presented in this study are available in supplementary materials and on request to the corresponding author.

## Supplementary Materials

The following are available online at https://www.mdpi.com/article/10.3390/v13091859/s1, Figure S1: PoAstV3 VPg sequences, Table S1: PoAstV3 case metadata, Table S2: Guanine-cytosine (GC) content of USA PoAstV3 identified in this study, Table S3: ORF1a/nsp1a nucleotide and amino acid percent identity matrix of PoAstV3, Table S4: ORF1ab/nsp1ab nucleotide and amino acid percent identity matrix of PoAstV3, Table S5: ORF2/capsid protein nucleotide and amino acid percent identity matrix of PoAstV3.
