# Supplementary material for: Comparative Analysis of Novel Strains of Porcine Astrovirus Type 3 in the USA
Source: Viruses. 2021 Sep 17;13(9):1859. doi: 10.3390/v13091859 (PMC8472076; doi:10.3390/v13091859)
Supplement: Supplementary file 1 [file viruses-13-01859-s001.zip › Supplemental materials viruses-1357937.pdf]

Supplemental materials: Results for DisoRDPbind webserver.

Further information regarding results formatting and interpretations can be found at: http://biomine.cs.vcu.edu. The annotations are encoded as follows: "1" denotes residues annotated with the particular type of binding, "0" denotes residues with annotations that include other types of disordered or ordered residues, and "x" denotes the residues that lack annotations

RNA-binding residues:

000000000000000000000000000000000000000000000000000000000000000000000000000000000000000000000
000000000000000000000000000000000000000000000000000000000000000000000000000000000000000000000
000000000000000000000000000000000000000000000000000000000000000000000000000000000000000000000
000000000000000000000000000000000000011111111111111111111111111111111111111111111111111111111
111111111111111110010000000000000000000000000000000000000000000000000000000000001000000000000
000000000000000000000000000000000000000000000000000000000000000000000000000000000000000000000
000000000000000000000000000000000000000000000000000000000000000000000000000111111111111110000
000000000000000000000000000000000000000000000000000000000000000000000000000000000000000000000
000000000000000000000000000000000000000000000000000000000000000101111000000000000000000000000
0000000

RNA-binding propensity:

0.092,0.058,0.052,0.058,0.057,0.051,0.052,0.058,0.060,0.062,0.071,0.061,0.068,0.068,0.075,0.072,0.073,0.085,0.073,0.077,0.077,0.086,0.091,0.087,0.082,0.079,0.069,0.072,0.073,0.083,0.065,0.071,0.073,0.072,0.055,0.053,0.055,0.053,0.043,0.042,0.053,0.064,0.058,0.054,0.057,0.064,0.066,0.064,0.053,0.044,0.038,0.046,0.043,0.039,0.044,0.042,0.060,0.058,0.058,0.064,0.066,0.065,0.080,0.094,0.096,0.077,0.096,0.085,0.092,0.092,0.072,0.066,0.056,0.051,0.055,0.052,0.047,0.043,0.048,0.055,0.072,0.091,0.081,0.073,0.059,0.081,0.079,0.081,0.085,0.113,0.111,0.102,0.098,0.117,0.095,0.096,0.091,0.109,0.112,0.108,0.089,0.099,0.110,0.119,0.102,0.087,0.081,0.106,0.106,0.077,0.068,0.063,0.063,0.065,0.064,0.080,0.069,0.057,0.052,0.047,0.056,0.044,0.045,0.045,0.044,0.065,0.058,0.065,0.074,0.073,0.054,0.046,0.048,0.045,0.039,0.031,0.030,0.031,0.030,0.032,0.026,0.023,0.021,0.022,0.018,0.019,0.016,0.019,0.020,0.017,0.018,0.014,0.012,0.014,0.011,0.010,0.010,0.011,0.012,0.018,0.021,0.020,0.020,0.014,0.019,0.023,0.024,0.023,0.022,0.018,0.016,0.021,0.020,0.021,0.021,0.022,0.029,0.027,0.032,0.031,0.029,0.022,0.024,0.020,0.019,0.022,0.019,0.018,0.016,0.018,0.018,0.014,0.012,0.010,0.011,0.011,0.013,0.013,0.010,0.012,0.015,0.019,0.019,0.017,0.021,0.020,0.027,0.030,0.028,0.037,0.029,0.027,0.024,0.024,0.020,0.024,0.019,0.021,0.025,0.025,0.017,0.017,0.020,0.020,0.019,0.018,0.015,0.016,0.013,0.011,0.011,0.010,0.012,0.008,0.007,0.008,0.009,0.011,0.016,0.016,0.020,0.024,0.027,0.026,0.022,0.020,0.022,0.027,0.032,0.032,0.042,0.034,0.035,0.047,0.046,0.047,0.046,0.041,0.049,0.044,0.046,0.042,0.039,0.034,0.040,0.052,0.041,0.039,0.044,0.051,0.040,0.046,0.049,0.056,0.041,0.044,0.049,0.043,0.048,0.052,0.053,0.047,0.052,0.058,0.079,0.075,0.080,0.084,0.095,0.095,0.096,0.097,0.072,0.071,0.074,0.070,0.069,0.085,0.089,0.111,0.099,0.108,0.112,0.137,0.135,0.109,0.096,0.103,0.115,0.128,0.126,0.117,0.139,0.138,0.145,0.150,0.158,0.185,0.228,0.238,0.216,0.251,0.248,0.214,0.223,0.262,0.243,0.227,0.199,0.253,0.249,0.231,0.258,0.282,0.270,0.245,0.240,0.263,0.244,0.227,0.235,0.238,0.261,0.266,0.265,0.305,0.318,0.354,0.283,0.325,0.353,0.357,0.335,0.333,0.352,0.397,0.415,0.428,0.448,0.470,0.475,0.488,0.498,0.482,0.500,0.550,0.523,0.454,0.468,0.414,0.396,0.380,0.328,0.314,0.336,0.343,0.343,0.319,0.333,0.279,0.245,0.263,0.263,0.297,0.230,0.263,0.267,0.174,0.155,0.149,0.130,0.155,0.144,0.134,0.135,0.119,0.101,0.072,0.071,0.077,0.081,0.067,0.056,0.066,0.070,0.079,0.065,0.071,0.073,0.074,0.058,0.061,0.050,0.045,0.038,0.045,0.032,0.032,0.033,0.035,0.031,0.033,0.041,0.038,0.047,0.056,0.049,0.056,0.060,0.037,0.041,0.049,0.041,0.042,0.040,0.046,0.038,0.032,0.037,0.049,0.044,0.062,0.081,0.085,0.102,0.091,0.084,0.099,

0.092,0.090,0.109,0.135,0.137,0.156,0.138,0.105,0.101,0.119,0.125,0.122,0.108,0.115,0.106,0.092,0.088,0.089,0.079,0.066,0.065,0.071,0.064,0.083,0.070,0.055,0.047,0.052,0.042,0.046,0.042,0.038,0.039,0.045,0.041,0.041,0.045,0.044,0.049,0.061,0.049,0.054,0.059,0.076,0.075,0.063,0.065,0.064,0.053,0.054,0.058,0.060,0.082,0.061,0.061,0.064,0.073,0.067,0.067,0.069,0.059,0.046,0.059,0.051,0.050,0.053,0.051,0.057,0.045,0.045,0.036,0.035,0.040,0.036,0.036,0.036,0.030,0.036,0.026,0.026,0.035,0.036,0.035,0.039,0.041,0.044,0.048,0.049,0.036,0.038,0.032,0.033,0.025,0.023,0.026,0.027,0.033,0.031,0.030,0.032,0.036,0.040,0.032,0.029,0.036,0.032,0.031,0.024,0.031,0.025,0.025,0.021,0.018,0.015,0.020,0.022,0.027,0.029,0.037,0.035,0.034,0.036,0.032,0.040,0.037,0.044,0.042,0.046,0.059,0.052,0.052,0.058,0.047,0.054,0.055,0.038,0.041,0.042,0.036,0.038,0.031,0.030,0.030,0.042,0.040,0.045,0.053,0.066,0.072,0.069,0.074,0.061,0.063,0.070,0.073,0.060,0.061,0.083,0.065,0.059,0.045,0.033,0.038,0.036,0.046,0.040,0.042,0.052,0.054,0.046,0.045,0.046,0.035,0.027,0.031,0.042,0.044,0.049,0.056,0.067,0.068,0.077,0.093,0.100,0.123,0.178,0.168,0.180,0.188,0.182,0.234,0.226,0.215,0.219,0.206,0.206,0.181,0.174,0.157,0.128,0.119,0.098,0.101,0.086,0.084,0.076,0.071,0.067,0.063,0.058,0.077,0.083,0.064,0.056,0.059,0.085,0.105,0.086,0.094,0.090,0.083,0.074,0.053,0.051,0.046,0.042,0.041,0.055,0.070,0.053,0.043,0.048,0.042,0.040,0.040,0.036,0.028,0.023,0.023,0.018,0.013,0.014,0.014,0.013,0.016,0.017,0.017,0.017,0.015,0.020,0.021,0.021,0.021,0.021,0.021,0.024,0.027,0.029,0.027,0.025,0.032,0.034,0.028,0.030,0.024,0.026,0.018,0.027,0.036,0.038,0.040,0.042,0.040,0.040,0.040,0.045,0.049,0.072,0.073,0.083,0.082,0.072,0.067,0.059,0.067,0.059,0.041,0.046,0.046,0.042,0.050,0.049,0.049,0.047,0.058,0.056,0.058,0.061,0.062,0.046,0.042,0.044,0.038,0.042,0.025,0.028,0.028,0.029,0.030,0.038,0.044,0.039,0.034,0.034,0.046,0.044,0.039,0.040,0.040,0.043,0.039,0.049,0.045,0.049,0.040,0.039,0.044,0.068,0.069,0.067,0.069,0.060,0.053,0.058,0.061,0.080,0.069,0.076,0.073,0.079,0.110,0.098,0.100,0.083,0.082,0.092,0.088,0.091,0.090,0.088,0.100,0.106,0.106,0.110,0.108,0.103,0.103,0.113,0.130,0.168,0.145,0.162,0.172,0.179,0.159,0.133,0.093,0.089,0.079,0.068,0.068,0.089,0.114,0.114,0.122,0.129,0.120,0.123,0.112,0.120,0.133,0.120,0.089,0.086,0.084,0.076,0.082,0.083,0.073,0.078,0.065,0.067,0.077,0.076,0.060,0.050,

DNA-binding residues:0000000000000000011111111111100000000000000000000000000000000100000000000000000000000000000000000000000000000000000000000000000000000000000000000000000000000000000000000000110001111111111111000000000000000000000000000000000000000000000000000000000000000000000000000000000000000000000000000000000000000000000000000000000000000000000000000000000000000000000000000000000000000001011111111111111111110110

DNA-binding propensity:

0.094,0.102,0.115,0.120,0.123,0.127,0.135,0.137,0.173,0.177,0.183,0.182,0.180,0.174,0.191,0.227,0.236,0.252,0.252,0.302,0.252,0.349,0.333,0.320,0.317,0.321,0.309,0.305,0.294,0.223,0.184,0.160,0.148,0.123,0.131,0.119,0.118,0.096,0.092,0.097,0.074,0.079,0.066,0.073,0.092,0.089,0.087,0.086,0.084,0.110,0.128,0.147,0.172,0.179,0.207,0.195,0.203,0.183,0.221,0.231,0.241,0.249,0.239,0.190,0.175,0.121,0.132,0.118,0.124,0.118,0.084,0.101,0.087,0.083,0.079,0.083,0.088,0.074,0.067,0.045,0.043,0.033,0.031,0.025,0.029,0.031,0.037,0.037,0.053,0.051,0.051,0.058,0.046,0.056,0.057,0.060,0.059,0.039,0.049,0.049,0.064,0.054,0.070,0.065,0.101,0.102,0.126,0.120,0.091,0.077,0.080,0.089,0.085,0.069,0.069,0.071,0.069,0.069,0.105,0.100,0.100,0.121,0.149,0.143,0.159,0.134,0.149,0.116,0.109,0.109,0.108,0.108,0.107,0.105,0.125,0.122,0.111,0.115,0.110,0.101,0.106,0.133,0.129,0.122,0.125,0.104,0.097,0.086,0.112,0.

124,0.151,0.142,0.145,0.139,0.145,0.146,0.153,0.168,0.166,0.181,0.190,0.196,0.199,0.184,0.184,0.181,0.230,0.281,0.281,0.226,0.231,0.222,0.231,0.226,0.226,0.231,0.196,0.198,0.188,0.182,0.167,0.160,0.150,0.139,0.141,0.144,0.176,0.160,0.130,0.140,0.146,0.122,0.106,0.103,0.099,0.068,0.056,0.056,0.051,0.041,0.041,0.036,0.036,0.034,0.033,0.036,0.033,0.033,0.033,0.031,0.028,0.028,0.035,0.029,0.028,0.031,0.038,0.046,0.057,0.060,0.071,0.075,0.088,0.093,0.097,0.099,0.097,0.097,0.069,0.072,0.076,0.084,0.059,0.053,0.082,0.083,0.078,0.091,0.093,0.094,0.096,0.082,0.067,0.060,0.060,0.061,0.059,0.059,0.050,0.054,0.052,0.052,0.044,0.068,0.071,0.068,0.073,0.073,0.076,0.073,0.073,0.068,0.074,0.095,0.132,0.133,0.128,0.134,0.133,0.163,0.167,0.175,0.178,0.196,0.181,0.152,0.158,0.145,0.151,0.146,0.154,0.153,0.161,0.170,0.162,0.091,0.090,0.098,0.103,0.103,0.102,0.110,0.115,0.101,0.097,0.102,0.124,0.118,0.130,0.124,0.089,0.085,0.085,0.085,0.089,0.089,0.130,0.130,0.102,0.093,0.093,0.098,0.091,0.089,0.100,0.085,0.088,0.084,0.081,0.077,0.081,0.105,0.110,0.105,0.105,0.106,0.110,0.112,0.108,0.130,0.137,0.138,0.108,0.107,0.105,0.104,0.126,0.100,0.088,0.082,0.081,0.078,0.088,0.087,0.095,0.100,0.100,0.092,0.076,0.053,0.053,0.053,0.043,0.056,0.056,0.056,0.056,0.057,0.062,0.077,0.087,0.087,0.091,0.091,0.087,0.079,0.061,0.061,0.064,0.076,0.091,0.113,0.113,0.112,0.106,0.111,0.113,0.108,0.102,0.117,0.139,0.139,0.140,0.110,0.104,0.109,0.109,0.139,0.145,0.139,0.139,0.118,0.093,0.078,0.120,0.120,0.122,0.101,0.087,0.090,0.085,0.065,0.067,0.055,0.073,0.075,0.090,0.092,0.091,0.083,0.088,0.086,0.131,0.128,0.143,0.117,0.121,0.115,0.132,0.152,0.130,0.137,0.144,0.136,0.163,0.153,0.129,0.099,0.098,0.091,0.097,0.101,0.105,0.095,0.101,0.088,0.091,0.090,0.093,0.081,0.075,0.089,0.087,0.083,0.085,0.087,0.093,0.115,0.121,0.119,0.126,0.118,0.091,0.084,0.085,0.104,0.123,0.114,0.076,0.074,0.089,0.100,0.096,0.096,0.090,0.095,0.115,0.107,0.107,0.115,0.126,0.123,0.124,0.146,0.187,0.188,0.150,0.155,0.132,0.187,0.187,0.179,0.172,0.175,0.143,0.159,0.147,0.114,0.114,0.108,0.106,0.091,0.095,0.099,0.103,0.103,0.133,0.137,0.111,0.129,0.132,0.126,0.129,0.135,0.136,0.158,0.166,0.171,0.146,0.141,0.155,0.150,0.168,0.162,0.170,0.169,0.139,0.112,0.106,0.106,0.113,0.111,0.113,0.113,0.108,0.106,0.117,0.109,0.104,0.114,0.119,0.113,0.107,0.101,0.106,0.085,0.087,0.090,0.095,0.102,0.131,0.123,0.123,0.120,0.101,0.106,0.106,0.103,0.110,0.113,0.126,0.126,0.126,0.139,0.141,0.130,0.130,0.121,0.118,0.093,0.086,0.083,0.089,0.090,0.094,0.109,0.109,0.108,0.102,0.079,0.078,0.064,0.060,0.058,0.052,0.067,0.089,0.111,0.117,0.119,0.118,0.120,0.114,0.111,0.090,0.090,0.096,0.094,0.123,0.104,0.127,0.108,0.132,0.139,0.149,0.132,0.103,0.087,0.087,0.073,0.075,0.075,0.075,0.075,0.076,0.076,0.075,0.072,0.077,0.062,0.077,0.078,0.096,0.100,0.080,0.063,0.093,0.097,0.091,0.090,0.101,0.076,0.094,0.103,0.106,0.101,0.118,0.097,0.095,0.088,0.088,0.109,0.112,0.137,0.112,0.169,0.244,0.215,0.228,0.237,0.237,0.256,0.307,0.313,0.355,0.411,0.435,0.460,0.516,0.523,0.429,0.441,0.330,0.276,0.192,0.201,0.201,0.135,0.126,0.117,0.091,0.109,0.107,0.086,0.089,0.089,0.075,0.069,0.066,0.069,0.066,0.101,0.096,0.096,0.118,0.144,0.167,0.142,0.185,0.153,0.126,0.148,0.114,0.092,0.114,0.114,0.088,0.079,0.066,0.070,0.054,0.058,0.057,0.046,0.054,0.044,0.029,0.030,0.027,0.025,0.033,0.039,0.041,0.035,0.045,0.043,0.033,0.036,0.030,0.025,0.019,0.025,0.023,0.024,0.020,0.017,0.020,0.031,0.037,0.037,0.037,0.044,0.037,0.029,0.035,0.032,0.031,0.037,0.035,0.042,0.062,0.082,0.080,0.103,0.080,0.143,0.156,0.155,0.150,0.117,0.123,0.109,0.088,0.106,0.136,0.175,0.148,0.126,0.132,0.138,0.151,0.125,0.117,0.146,0.096,0.097,0.097,0.105,0.098,0.099,0.099,0.122,0.136,0.111,0.115,0.108,0.085,0.100,0.152,0.146,0.140,0.129,0.151,0.153,0.153,0.131,0.157,0.122,0.133,0.149,0.127,0.126,0.097,0.097,0.116,0.113,0.092,0.077,0.079,0.053,0.054,0.053,0.037,0.031,0.031,0.024,0.031,0.032,0.034,0.033,0.022,0.021,0.028,0.031,0.030,0.031,0.030,0.031,0.036,0.035,0.044,0.058,0.073,0.105,0.156,0.159,0.195,0.226,0.212,0.257,0.214,0.305,0.413,0.361,0.357,0.347,0.404,0.427,0.486,0.488,0.496,0.472,0.398,0.330,0.347,0.299,0.297,0.247,0.252,0.269,0.210,0.269,0.260,0.188,

Protein-binding residues:

0000000000000000000000000000000000000000000000000000000000000000000000000000000000000000
0000000000000000000000000000000000000000000000000000000000000000000000000000000000000000
0000000000000000000000000000000000000000000000000000000000000000000000000000000000000000
0000000000000000000000000000000000000000000000000000000000000000000000000000000000000000

00000000000000000000000000000000000000000000000000000000000000000000000000000000000000000000
00000000000000000000000000000000000000000000000000000000000000000000000000000000000000000000
00000000000000000000001000000000000110000000000000000000000000000000000000000000000000000000
00000000000000000000000000000000000000000000000000000000000000000000000000000000000000000000
00000000000000000000000000000000000000000000000000000000000000000000000000000000000000000000
0000000

Protein-binding propensity:

0.391,0.372,0.356,0.358,0.398,0.423,0.415,0.371,0.326,0.278,0.293,0.267,0.262,0.256,0.250,0.263,0.264,0.283,0.31
0,0.318,0.314,0.293,0.301,0.325,0.356,0.388,0.399,0.390,0.388,0.405,0.409,0.426,0.420,0.413,0.424,0.415,0.427,0.
451,0.428,0.452,0.441,0.444,0.462,0.486,0.466,0.439,0.449,0.449,0.458,0.449,0.418,0.448,0.438,0.448,0.466,0.454,
0.428,0.388,0.377,0.383,0.382,0.401,0.387,0.392,0.399,0.369,0.391,0.380,0.392,0.379,0.364,0.383,0.392,0.434,0.45
3,0.433,0.450,0.479,0.478,0.474,0.460,0.428,0.416,0.435,0.401,0.407,0.403,0.405,0.407,0.416,0.393,0.403,0.410,0.
426,0.419,0.423,0.426,0.423,0.425,0.388,0.415,0.390,0.396,0.392,0.361,0.359,0.347,0.361,0.336,0.343,0.371,0.350,
0.358,0.378,0.372,0.429,0.422,0.425,0.412,0.427,0.444,0.440,0.494,0.481,0.468,0.451,0.433,0.429,0.444,0.462,0.48
0,0.489,0.478,0.487,0.503,0.494,0.493,0.488,0.482,0.457,0.471,0.484,0.462,0.457,0.435,0.420,0.450,0.439,0.404,0.
408,0.406,0.401,0.380,0.354,0.351,0.364,0.345,0.344,0.348,0.349,0.365,0.338,0.320,0.316,0.326,0.321,0.315,0.312,
0.324,0.333,0.315,0.330,0.300,0.293,0.293,0.317,0.322,0.328,0.344,0.350,0.341,0.370,0.384,0.394,0.389,0.400,0.44
1,0.442,0.463,0.471,0.482,0.477,0.488,0.462,0.475,0.487,0.487,0.483,0.510,0.503,0.503,0.507,0.502,0.476,0.497,0.
485,0.455,0.472,0.472,0.526,0.524,0.508,0.508,0.504,0.477,0.484,0.486,0.493,0.483,0.424,0.394,0.376,0.374,0.362,
0.340,0.348,0.357,0.362,0.340,0.333,0.317,0.303,0.286,0.277,0.283,0.323,0.347,0.367,0.397,0.395,0.350,0.343,0.32
0,0.332,0.341,0.349,0.358,0.336,0.330,0.320,0.338,0.329,0.324,0.295,0.285,0.272,0.310,0.306,0.318,0.303,0.317,0.
331,0.333,0.299,0.307,0.323,0.321,0.320,0.320,0.301,0.284,0.294,0.298,0.314,0.317,0.322,0.299,0.283,0.262,0.258,
0.260,0.264,0.255,0.245,0.264,0.309,0.313,0.332,0.335,0.318,0.309,0.308,0.316,0.271,0.281,0.295,0.306,0.313,0.30
0,0.276,0.284,0.285,0.285,0.290,0.271,0.304,0.287,0.286,0.286,0.312,0.283,0.297,0.292,0.292,0.297,0.297,0.309,0.
301,0.297,0.294,0.282,0.277,0.302,0.305,0.296,0.282,0.299,0.267,0.287,0.286,0.259,0.284,0.292,0.308,0.326,0.302,
0.301,0.278,0.266,0.265,0.259,0.273,0.263,0.309,0.272,0.258,0.285,0.285,0.278,0.262,0.254,0.281,0.298,0.293,0.28
8,0.257,0.274,0.274,0.291,0.275,0.300,0.299,0.299,0.302,0.321,0.310,0.279,0.292,0.292,0.289,0.292,0.315,0.313,0.
314,0.299,0.311,0.279,0.286,0.288,0.282,0.288,0.270,0.269,0.287,0.278,0.267,0.249,0.266,0.262,0.252,0.266,0.269,
0.320,0.305,0.322,0.349,0.351,0.358,0.385,0.418,0.400,0.419,0.431,0.427,0.424,0.428,0.429,0.463,0.460,0.495,0.49
9,0.501,0.484,0.469,0.470,0.483,0.447,0.441,0.410,0.404,0.417,0.413,0.443,0.454,0.423,0.407,0.404,0.371,0.386,0.
396,0.389,0.371,0.354,0.356,0.401,0.391,0.361,0.368,0.371,0.365,0.378,0.405,0.382,0.373,0.365,0.377,0.405,0.393,
0.399,0.409,0.410,0.465,0.469,0.463,0.467,0.431,0.399,0.389,0.402,0.412,0.405,0.403,0.388,0.395,0.405,0.397,0.40
3,0.385,0.383,0.364,0.350,0.348,0.360,0.327,0.320,0.321,0.339,0.375,0.420,0.410,0.441,0.446,0.443,0.491,0.475,0.
473,0.486,0.493,0.500,0.521,0.516,0.486,0.494,0.506,0.508,0.522,0.493,0.488,0.498,0.487,0.483,0.518,0.529,0.549,
0.556,0.523,0.528,0.526,0.535,0.527,0.491,0.475,0.482,0.478,0.471,0.468,0.443,0.443,0.415,0.411,0.418,0.444,0.45
1,0.468,0.488,0.475,0.491,0.475,0.472,0.489,0.486,0.465,0.477,0.472,0.505,0.520,0.516,0.477,0.500,0.508,0.516,0.
505,0.514,0.502,0.505,0.512,0.500,0.537,0.577,0.548,0.561,0.552,0.531,0.535,0.526,0.531,0.519,0.505,0.510,0.470,
0.468,0.442,0.441,0.456,0.459,0.476,0.479,0.506,0.491,0.494,0.500,0.494,0.516,0.521,0.531,0.556,0.589,0.600,0.59
3,0.602,0.801,0.780,0.780,0.569,0.571,0.585,0.548,0.553,0.550,0.776,0.791,0.591,0.612,0.801,0.809,0.626,0.634,0.
615,0.795,0.798,0.777,0.769,0.791,0.793,0.585,0.552,0.767,0.526,0.517,0.752,0.749,0.730,0.727,0.717,0.727,0.728,
0.423,0.712,0.408,0.402,0.396,0.426,0.415,0.401,0.694,0.371,0.684,0.669,0.652,0.653,0.317,0.357,0.683,0.361,0.36
2,0.366,0.360,0.358,0.363,0.353,0.351,0.321,0.304,0.657,0.327,0.661,0.325,0.330,0.667,0.676,0.343,0.372,0.375,0.
371,0.676,0.677,0.317,0.668,0.376,0.389,0.393,0.393,0.408,0.423,0.704,0.384,0.688,0.381,0.684,0.363,0.700,0.695,

0.417,0.424,0.407,0.415,0.453,0.463,0.469,0.739,0.494,0.751,0.513,0.764,0.773,0.535,0.770,0.764,0.529,0.778,0.55
4,0.556,0.536,0.562,0.543,0.550,0.761,0.760,0.781,0.778,0.778,0.584,0.600,0.592,0.626,0.605,0.641,0.625,0.641,0.
618,0.601,0.595,0.602,0.591,0.570,0.554,0.552,0.559,0.581,0.597,0.581,0.578,0.559,0.545,0.532,0.561,0.586,0.564,
0.572,0.530,0.531,0.506,0.513,0.471,0.450,0.462,0.447,0.428,0.437,0.422,0.430,0.428,0.412,0.409,0.401,0.436,0.40
6,0.407,0.434,0.432,0.421,0.408,0.417,0.403,0.407,0.402,0.384,0.433,0.404,0.389,0.390,0.392,0.391,0.363,0.377,0.
407,0.419,0.425,0.415,0.417,0.387,0.375,0.390,0.365,0.381,0.397,0.385,0.371,0.327,0.351,0.356,0.362,0.328,0.340,
0.358,0.314,0.338,0.347,0.349,0.338,0.319,0.311,0.312,0.296,0.317,0.334,0.339,0.329,0.326,0.306,0.302,0.349,0.33
3,0.349,0.346,0.326,0.321,0.311,0.301,0.321,0.319,0.330,0.361,0.346,0.348,0.360,0.352,0.361,0.401,0.407,0.385,0.
400,0.398,0.408,0.427,0.436,0.432,0.419,0.426,0.416,0.410,0.424,0.435,0.402,0.374,0.448,0.467,0.450,0.472,0.485,
0.499,0.516,0.504,0.575,

>ORF1a Consensus sequence (1-845). Protein encoded sequence, where the lower (upper) case indicates the residue was predicted to interact (not to interact) with RNA/DNA/protein

MALPFDDTLKFGSAAARvkgrrldsvartKLKDLLGDGPYFYDFGPLEVVDSSSKQLTVKMtSVQTVYVSSVV
EDNSYVTYKFVPGVNEWVETEPVLHKPTALVGVLWREYNRYKDKTESLSQELSQLRLEHSLLRHDYER
VRPQQPATPRCRLGFITKLLIGLLIGslMAHSTLAHKTPGTGLLGECLDTDVIDGKQVCVNFLPWNETET
NPTDGDKTTTISWDHEGLLDLLVLIAPYLVSWPMIATMVGFFYVATAEQPAYMLVSLLLATYSKAQVL
ALAGLPFMDMPSVVTLWVSMLVHSYDAVLSLWVALLLaaftlsagvfmpdvrysdlvrgqlvvflvlvfnylvvmmsl
pnwlvfslvvgyrvlrvltflvaekvevrgpdGKvVETRTSMPAWINKASNFLQTRFHQKIRTGISPTARVIPNGVIVV
ETQDGSGTGFRCRNYLVtAGHVVTGTETPKIQWAGVTAYSKVVHRVPDKDIAFLAIPQELQDLPSYRLA
KKVEDGPVVITSLEDSGALSVAVTEGVIVTDSITYAVQTRNGMSGSPVTNLDGRILAVHQTNTGFTGGA
VILTDEdFPQPKKSAREQQleARIKELEAAMNQSSNHEDIVELVRAAVAREFKVLRTELsattfiqa**kgknkk**h
hrrrggKKRRAVW**SEEEY**KELLEKGFSKSQLRDMAEVLRSHEEDPFGSDTESEGGFPEWSDVSDAESIERE
WFGQSWEDCKPVKEEPQDTLPVHLKEKYTLDAYVISKEELRSFAKEFKDYVDKVEALIDKTVQQGKWL
PSVNPTAIIEELNDLWFGLNmLmwekGLVPFTqRkkikrkvqknskgapkrgpLptP

Three non globular protein disordered regions and three putative disordered RNA-protein binding regions were identified within the nsp1a with DisoRDPbind webserver, respectevely. Protein disordered regions were identified betwwen residues 206-222 (consensus WNETETNPTDGDKTTTI), 691- 709 (consensus EDPFGSDTESEGGFPEWSD) and 832-845 (consensus SKGAPKRGPLPTP). Putative disordered RNA-protein bindig regions were located within residues 316-392 (AAFTLSAGVFMPDVRYSDLVRGQLVVFLVLVFNYLVVMMSLPNWLVFSLVVGYRVLRVLTFLVAEKV EVRGPDGKV), 633- 647 (SATTFIQA**KGK**NKK) and residues 807-813(MLMWEK).
