# Supplementary figures and images for: Comparative Analysis of Novel Strains of Porcine Astrovirus Type 3 in the USA
Source: Viruses. 2021 Sep 17;13(9):1859. doi: 10.3390/v13091859 (PMC8472076; doi:10.3390/v13091859)

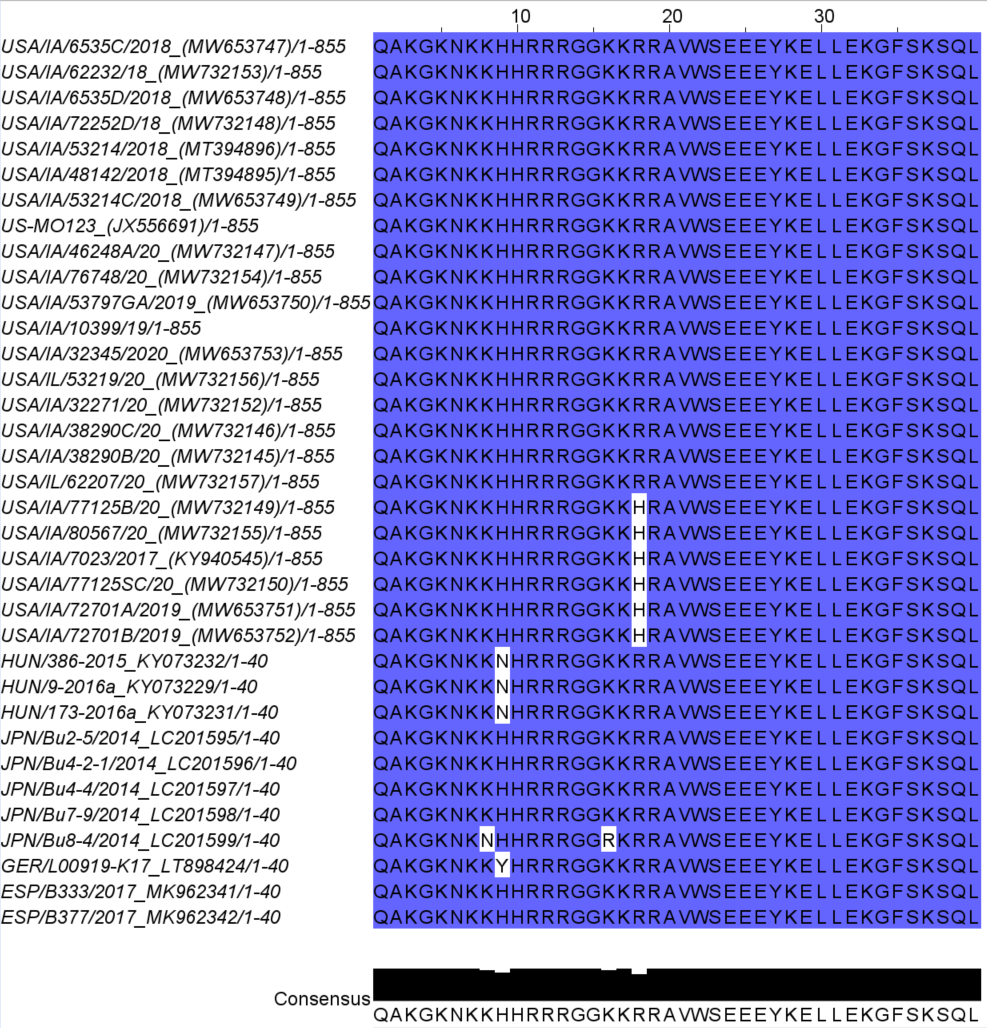

Supplement: Supplementary file 1 [file viruses-13-01859-s001.zip › Supplemental Figure S1- VPg consensus 600 dpi.tif]
